# Eating disorders among patients incarcerated only for repeated shoplifting: a retrospective quasi-case-control study in a medical prison in Japan

**DOI:** 10.1186/1471-244X-14-169

**Published:** 2014-06-07

**Authors:** Tomokuni Asami, Yoshiro Okubo, Mizuho Sekine, Toshiaki Nomura

**Affiliations:** 1 Department of Psychiatry, Hachioji Medical Prison Hospital, 2-26-1 Koyasu, Hachioji, Tokyo 192-0904, Japan; 2 Psychiatry Department, Nippon Medical School, 1-1-5 Sendagi, Bunkyo-ku, Tokyo 113-8602, Japan; 3 Psychology Department, Nippon Medical School, 1-396 Kosugi-chou, Nakahara Kawasaki, Knagawa 211-8533, Japan

**Keywords:** Anorexia nervosa, Shoplifting, Multi-impulsive bulimia nervosa, Crime, Addiction

## Abstract

**Background:**

Shoplifting is a serious problem among patients with eating disorders. For more than a decade, we have treated many patients with eating disorders incarcerated in Hachioji Medical Prison only for repeated shoplifting.

**Methods:**

We analyzed the prison records and medical records of female psychiatric patients transferred to Hachioji Medical Prison between 2002 and 2011. Based on the offense listed at the time of sentencing, we extracted a shoplifting group and a drug-offense group from among all patients with eating disorders. One patient from the former group who had used substances and two from the latter group who had never shoplifted were excluded from the study. The groups had 41 and 14 patients, respectively. A control group comprised patients with other mental disorders (n = 34). We compared eating disorder histories and subtypes, weight changes, comorbidities, life histories, past behavioral problems, and clinical behavioral problems among the three groups.

**Results:**

The shoplifting group exhibited less impulsive behavior, substance abuse, antisocial features, borderline personality disorder, and past bulimia than did the drug-offense and control groups. The shoplifting group had higher educational achievement and steadier employment; however, their eating disorder histories and interpersonal dysfunction were more severe, and they had a higher psychiatric treatment dropout rate. There were also significant relationships with low body weight, anorexia nervosa-restricting type, obsessive–compulsive behaviors, and obsessive–compulsive personality disorder in the shoplifting group. During the clinical course, food refusal, excessive exercise, food hoarding, and falsification of dietary intake amounts were more frequently observed in the shoplifting group. Conversely, drug requests and occurrences of self-harm were less frequent in the shoplifting group than in the drug-offense group.

**Conclusions:**

Although these results may be associated with specific characteristics of patients with eating disorders in the medical prison setting, we concluded that the repeated shoplifting by these patients is unrelated to antisocial or impulsive characteristics but is deeply rooted in these patients’ severe and undertreated eating disorder psychopathology. Strong supportive treatment should be considered for patients with eating disorders who develop shoplifting behaviors. Further research is required to elucidate the mechanisms responsible for the relationship between shoplifting and eating disorders.

## Background

Shoplifting is one of the more serious behavioral problems that develop among patients with eating disorders. However, the reasons for the association between shoplifting and eating disorders remain unclear. For more than a decade, we have managed many patients with eating disorders who continued to engage in repeated shoplifting until they were incarcerated. Imprisonment is a consequence of repeated shoplifting. Currently, there are no established treatment programs to reduce the incidence of shoplifting among patients with eating disorders [1]. The association between shoplifting and eating disorders has been reported for more than 30 years [2-5]. Shoplifting behavior in patients with eating disorders is increasingly being encountered in clinical practice in Japan, and awareness of this problem is growing among specialists and lawyers who have worked with these patients. Some eating disorder experts have defended patients prosecuted for shoplifting in court, asserting that treatment should take priority over punishment [6,7]. However, we believe that treatment without legal prosecution may merely delay the incarceration of these patients.

Because shoplifting is considered a misdemeanor, exemption from prosecution is possible in many cases at every stage after the offense, including at the store where it occurred, the police station, the prosecutor’s office, or even at trial if the patient promises to receive treatment. Defendants are typically only sentenced to a prison term after two or more probation sentences (i.e., they continued to shoplift despite multiple arrests, eventually leading to incarceration). When a person is about to be incarcerated for shoplifting of food only, he or she must have been engaging in repeated shoplifting or exhibiting difficulty correcting this seriously problematic behavioral pattern. That is, it can be said that the shoplifting behavior no longer appears to be merely an impulsive behavior.

According to a survey by the Japanese Association of Correctional Medicine, 95 of the 4,148 inmates in women’s prisons in Japan had been diagnosed with an eating disorder as of 15 October 2011 [8]. Inmates in any of the 185 correctional facilities in Japan displaying highly refractory illness or who pose a potential physical risk are often transferred to a medical prison. Three such national specialty hospitals are operated by the Ministry of Justice. Emergent requests for transfer of patients with eating disorders to the Hachioji Medical Prison have increased over the last decade [8]. Eating disorders have been the most critical ailments (mental or physical) encountered in correctional facilities in Japan over the last 5 years, with many patients being incarcerated for shoplifting or drug offenses [8]. Although this medical prison has a relatively high degree of treatment, the patients are placed in nearly the same restrictive environment as a general prison in Japan.

A significant positive association has been reported between shoplifting and antisocial behavior; shoplifting has also been strongly associated with disorders encompassing impulse control deficits, including antisocial personality disorder, substance use disorders, pathological gambling, and bipolar disorder [9]. Moreover, shoplifting has often been associated with the impulsive behaviors of patients with bulimia nervosa (BN) or bulimic behaviors [10-16]. In particular, shoplifting is regarded as a key symptom of multi-impulsive BN [11-14,16-18]. One study noted that patients with anorexia nervosa (AN) of the binge–purge type were more likely to attempt stealing than were patients with restricting-type AN (AN-R) [13]. In addition, patients with a personal childhood history of physical or sexual abuse typically experience more alcohol problems, suicide attempts, and shoplifting than do patients without such experiences [19]. Borderline personality disorder has been correlated with impulsive behaviors, including shoplifting, by patients with AN [20]. Shoplifters with eating disorders may demonstrate low self-esteem, increased depression, and purging behaviors at the time of assessment [21]. Furthermore, shoplifting is argued to be a factor related to prolonged inpatient treatment of patients with AN [22]. Indeed, previous studies have suggested that shoplifting behavior may define a more severe eating disorder subtype [10,12,22-25].

Many studies have suggested that shoplifting among patients with eating disorders is correlated with antisocial features and/or impulsive behaviors [2,3,11-24]. However, previous studies have not typically considered the severity or frequency of shoplifting in such patients. Moreover, we found no evidence for patients with eating disorders who were actually incarcerated for shoplifting. In our facility, patients with eating disorders who were incarcerated for offenses other than shoplifting sometimes confess to shoplifting. However, these patients rarely have a history of an actual prison sentence for shoplifting [8]. We did not find any reports distinguishing between those with a history of other illegal behaviors and those incarcerated only for shoplifting, among patients with eating disorders in the medical prison. The present study was conducted to examine the characteristics of patients with eating disorders who were incarcerated for repeated shoplifting and to clarify the clinical significance of this specific behavioral problem in this group.

## Methods

### Study design and participants

This was a retrospective quasi-case-control study based on the structured prison records and medical records of female patients transferred to the Hachioji Medical Prison Hospital from general prisons for psychiatric treatment from October 2002 to December 2011. We investigated 131 consecutively admitted female patients throughout the study period. Of these, 70 were diagnosed with eating disorders at the time of transfer to the hospital (age range, 20–62 years). Sufficient information was available to categorize 67 of these patients (age range, 20–50 years). These 67 patients were classified based on the conviction for which they were incarcerated. The most common conviction resulting in imprisonment was shoplifting (*n* = 42; 63%). No patients in this group had a history of conviction for other crimes. The second most common conviction resulting in imprisonment was a drug-offense (*n* = 16; 24%); no patients who had committed drug offenses had an incarceration history for shoplifting. The main convictions among the remaining nine patients with eating disorders were arson (*n* = 2; 3%), murder (*n* = 2; 3%), and other crimes (*n* = 5; 7%). Only one of these nine patients had a history of being sentenced for shoplifting. However, no overlap in the judgment of the crime was present between patients incarcerated only for shoplifting and those incarcerated for drug -offenses. To compare the characteristics of behavioral problems among homogeneous populations of patients with eating disorders, we looked for overlapping misconduct between the two eating disorder groups. There was one patient who had history of substance-use among the 42 patients incarcerated only for shoplifting, and we excluded this one patient. Two patients among the 16 patients in the drug-offense group did not have a history of shoplifting, and we excluded these two patient. The 41 patients incarcerated for shoplifting were classified into the Shoplifting (S) group, and the 14 patients incarcerated for drug-related offenses were classified into the drug-offense (D) group. We decided to investigate these two groups. Thus, this is a comparison of patients with eating disorders who were incarcerated only for shoplifting, and patients with eating disorders who were incarcerated for drug-related offenses and also had a history of shoplifting. The remaining patients with eating disorders were excluded from the analysis. The control (C) group comprised female patients with other psychiatric disorders who were matched with patients in the S group for study duration and age (n = 34).

### Medical records and diagnosis

We collected data on diagnosis, comorbidities, life history, eating disorder history, addictions, and behavioral problems from the medical records. Variables other than body mass index (BMI), education, and eating disorder history were recorded on a dichotomous yes/no scale. We defined repeated shoplifting to be habitual, relentless, and ruinous shoplifting that continued despite fines or arrests.

### Diagnosis and measures assessing life history

Psychiatric disorders and comorbidities were diagnosed based on the Diagnostic and Statistical Manual of Mental Disorders, Fourth Edition, Text Revision. Primary diagnoses were confirmed by at least two to three experienced psychiatrists (T.A., M.S., and T.N.) within 14 days of hospital admission, and comorbidities were identified throughout the treatment course. Obsessive–compulsive disorder (OCD) was diagnosed by excluding symptoms associated with eating disorders, such as excessive exercise, anal disimpaction, laxative abuse, compulsive buying, and food hoarding. The two eating disorder groups were compared in terms of their history of eating disorders, ratio of eating disorder subtypes, past BN history, and comorbidities. The characteristics of the two eating disorder groups and the C group were compared with respect to age distribution, BMI at imprisonment and hospital admission, treatment history, life history (family environment, education, delinquency, occupation, marriage, and other interpersonal relationships), and past behavioral problems.

### Clinical observations

Behavioral problems interfering with the recovery of patients with excessively low body weight were monitored after their admission to the hospital. Data were recorded by nurses based on their observations within a strict 24-h surveillance-equipped environment within the medical prison. Patients were considered to have remarkable symptoms if they exhibited disruptive behavior (use of violent language, frequent drug requests, self-harm, food refusal, purging, falsifying dietary intake amounts, food hoarding, or excessive exercise). These patterns of behavior were summarized using a Likert-type scale with the variable “exhibiting the behavior”: 1, never; 2, sometimes; 3, almost every day; and 4, several times a day, and levels 3 and 4 were considered disruptive. Excessive exercise (i.e., obstinate engagement in repetitive physical exercise) was defined as exercising outside permitted exercise times or in secret despite repeated warnings from nurses to stop.

Each patient stayed in a single room, and all rooms were locked by prison officials. Because these patients continued to engage in many prohibited behaviors, it was often difficult to monitor all of these behaviors. Thus, the following measures were enforced when more accurate observation was needed in the course of treatment: inspection of belongings (toilet seats, garbage boxes, bedding, and clothes), restriction of faucets and drain outlets (water use was not allowed without observation by staff), and food intake in a separate room with 2-h postprandial monitoring.

### Statistical analyses

The parametric variables eating disorder history, treatment duration, and BMI were analyzed in the two groups using unpaired t-tests. Analysis of variance (ANOVA) was used for comparisons of the age distribution, education level, and BMI among the three groups; significant ANOVA results were followed by post-hoc tests with Bonferroni correction for multiple analyses. Descriptive data were reported as mean ± standard deviation and *n* (%). Significant differences in dichotomous variables between two or among three groups were explored using the χ^2^ test, the Yates exact test (if *n* < 10), or Fisher’s exact test (if *n* < 4). The significance level was set at 0.05.

### Ethical approval

The study protocol was approved by the Medical Ethics Committee of the Japanese Association of Correctional Medicine. Because this was a retrospective study using medical records from Hachioji Medical Prison Hospital and not an interventional study, we did not obtain written informed consent from the patients as per the Ethical Guidelines for Clinical Research of the Ministry of Health, Labour and Welfare of Japan, revised 31 July 2008.

## Results

### Patient characteristics, illness history, body weight, diagnosis, and comorbidities (Table 1)

**Table 1:** Patient characteristics, illness history, body weight, diagnosis, and comorbidities

	**Shoplifting (S) Group (*n*** **= 41)**	**Drug-offense (D) Group (*n*** **= 14)**	**Control (C) Group (*n*** **= 34)**	**TS**	***p***
	**Mean (s. d.)**	**Mean (s. d.)**	**Mean (s. d.)**		
Age [years]	37.1^a^ (7.4)	29.9^b^ (6.3)	32.9^b^ (6.7)	*F* = 6.7	<0.002*
Range	24–50	23–45	23–48		
Eating disorder history [years]	15.7 (6.7)	6.7 (5.9)	NA	T = 4.8	<0.001**
Range	5–31	0–16	-		
BMI (body mass index) at imprisonment [kg/m^2^]	13.6^a^ (1.9)	17.6^b^ (3.1)	23.0^c^ (4.4)	*F* = 77.0	<0.001*
Range	10.5–19.1	13.0–21.9	17.2–40.3		
BMI at admission [kg/m^2^]	11.6 (0.8)	11.5 (0.7)	-	T = 1.6	*0.06**
Range	9.6–14	10.8–14.3	-		
	*n* (%)	*n* (%)	*n* (%)		
Treatment history for psychiatric disease	29 (71)	5 (36)	24 (71)	4.7	0.10***
Outpatient	9 (22)	1 (7)	6 (18)	0.74	0.69***
Hospitalization	20 (49)	4 (29)	18 (53)	1.5	0.47***
Continued until latest arrest	8 (20)	1 (7)	4 (12)	0.67	0.71***

Significance level: *p* < 0.05.

Post-hoc analyses for significant differences (*χ*^2^ test: *p* < 0.05) are indicated by superscript letters “a” and “b*.*” Groups sharing a letter did not significantly differ.

*Non-repeated–measures analysis of variance (ANOVA). Significant ANOVA results were followed by post-hoc tests with Bonferroni’s correction for multiple analyses. Degree of freedom (df): first df = 2, second df = 86.

**Non-paired *t*-test: df = 53. Tests were two-tailed.

***Statistical tests used were the *χ*^2^ test, or Yates exact test for dichotomous variables: df = 2.

Patients in the S group were significantly older than those in the D and C groups. The history of eating disorders was markedly long in the S group (15.7 ± 6.7 years). One patient did not have an eating disorder history before incarceration. However, her clinical symptoms in the prison met the diagnostic criteria of the DSM-4 TR, so we included this patient in the D group in this study.

The BMI at the time of imprisonment was significantly lower in the S group (13.6 ± 1.9 kg/m^2^) than in the D (17.6 ± 3.1 kg/m^2^) and C groups (23.0 ± 4.4 kg/m^2^). There were more patients with a history of psychiatric treatment in the S group (*n* = 29; 71%) than in the D group (*n* = 5; 36%). Few patients were undergoing treatment just before their most recent arrest (8 [20%] in the S group).

### Main diagnoses, axis II disorders, and comorbidities (Table 2)

**Table 2:** Main diagnosis, axis II disorders, and comorbidities

	**Shoplifting (S) Group (*n*** **= 41)**	**Drug-offense (D) Group (*n*** **= 14)**	**Control (C) Group (*n*** **= 34)**	**TS**	***P***
	***n *** **(%)**	***n *** **(%)**	***n *** **(%)**		
Axis I				[*χ*^2^-test for S, D]	
ANR: restricting-type anorexia nervosa	12 (29)	0	-	-*	0.022
ANBP: binge–purge anorexia nervosa	29 (71)	14 (100)	-	-	-
Past bulimia nervosa history	7 (17)	9 (64)	-	9.1	0.003
Schizophrenia	0	0	13 (38)	-	-
Mood disorder	15 (37)	9 (64)	1 (3)	2.2	0.14
Stimulant psychosis	0	9 (64)	9 (21)	*	<0.001
Reaction psychosis	5 (12)	2 (14)	6 (18)	*	0.79
Pervasive developmental disorders	0	0	2 (6)	-	-
Mental retardation	0	0	2 (6)	-	-
Obsessive–compulsive disorder	13 (32^a^)	0^b^	1 (3^b^)	*	0.04
Other anxiety disorders	9 (22)	2 (14)	0	*	0.82
Somatoform disorder	3 (7)	1 (7)	3 (9)	*	0.57
Impulse control disorder not elsewhere classified	1 (Kleptomania)	1 (Pyromania)	0	-	-
Axis II				[*χ*^2^-test for 3 groups]	
Borderline personality disorders (PD)	4 (10^a^)	9 (64^b^)	13 (33^c^)	14.3	<0.001
Antisocial PD	0 (0^a^)	4 (29^b^)	7 (21^b^)	8.2	0.017
Obsessive compulsive PD	8 (20^a^)	0^b^	0^b^	7.2	0.027

Significance level: *p* < 0.05.

Statistical tests used were the *χ*^2^ test, Yates exact test, or Fisher’s exact test for dichotomous variables. Post-hoc analyses for significant differences (*χ*^2^ test: *p* < 0.05) are indicated by superscript letters “a” and “b*.*” Groups sharing a letter did not significantly differ. Degree of freedom: *χ*^2^ test for all three groups, 2; *χ*^2^ test for two groups, 1. *Data could not be detected from Fisher’s exact probability.

Among the subtypes, AN-R was observed only in the S group (*n* = 12; 29%). The number of patients with a history of crossover between AN and BN was 7 (17%) in the S group and 9 (64%) in the D group. Comorbid OCD occurred in 13 (32%) patients in the S group, but in none in the D group. One patient in the S group was diagnosed with impulse-control disorder not otherwise classified (kleptomania). Fewer patients were diagnosed with borderline personality disorder in the S group (*n* = 4; 10%) than in the D group (*n* = 9; 64%). Comorbid OC personality disorder was only observed in the S group (*n* = 8; 20%).

### Clinical background and life history (Table 3)

**Table 3:** Clinical background and life history

	**Shoplifting (S) Group (*n*** **= 41)**	**Drug-offense (D) Group (*n*** **= 14)**	**Control (C) Group (*n*** **= 34)**	**TS**	***p***
	**N (%)**	**N (%)**	**N (%)**		
Family environment					
Anti-social feature	0 (0^a^)	6 (43^b^)	8 (24^c^)	13.4	0.001
Poverty	4 (10^a^)	11 (79^b^)	11 (32^c^)	20.1	<0.001
Family breakup	9 (22^a^)	10 (71^b^)	13 (38^a^)	9.1	0.011
Alcoholism trend	6 (15)	6 (43)	10 (29)	3.5	0.17
Infantile abuse (physical, sexual)	1 (2^a^)	7 (50^b^)	6 (17^b^)	14.5	<0.001
Juvenile delinquency (arrest) history	0 (0)	12 (86^b^)	15 (44^c^)	36.7	<0.001
Experience as regular employee	39 (95^a^)	3 (21^b^)	18 (53^b^)	27.2	<0.001
Prostitution as primary job	2 (5^a^)	8 (57^b^)	14 (41^b^)	16.9	<0.001
Marriage history rate	20 (49)	11 (79)	18 (53)	2.7	0.26
Divorce	10 (24^a^)	8 (57^b^)	11 (61^b^)	3.7	0.15
Connection with antisocial person	0 (0^a^)	11 (79^b^)	12 (35^c^)	31.8	<0.001
Interpersonal relation other than parents (maintained for one year until their arrests)	13 (32^a^)	13 (93^b^)	28 (82^b^)	23.9	<0.001
	Mean (s. d.)	Mean (s. d.)	M (s. d.)		
Education years	13.1^a^ (1.7)	9.6^b^ (0.9)	9.9^b^ (1.5)	53.8*	<0.001*
Range	9–16	9–12	9–16		

Significance level: *p* < 0.05.

Statistical tests used were the *χ*^2^ test or Yates exact test for dichotomous variables. Post-hoc analyses for significant differences are indicated by superscript letters “a,” “b,” and “c”; groups sharing a letter did not significantly differ. Degree of freedom (df): *χ*^2^ test for all three groups, 2.

*Non-repeated–measures analysis of variance (ANOVA). Test was two-tailed. Significant ANOVA results were followed by post-hoc tests with Bonferroni’s correction for multiple analyses: first df = 2, second df = 86.

The home environments of patients in the S group were not characterized by higher incidences of antisocial features, poverty, family disintegration, or infantile abuse than the other groups. The education level of patients in the S group (mean, 13.1 years) was markedly higher than that of patients in the other groups: 7 patients were university graduates, 15 were junior college or technical school graduates, and some were qualified nurses, nutritionists, law officials, or teachers. Regular employment was more common in the S group than in the other two groups, with 39 (95%) patients having established lifelong regular employment. The proportion of patients engaged in prostitution was significantly lower in the S group (*n* = 2; 5%) than in the other two groups. There was no history of companionship with antisocial individuals in the S group. Fewer patients in the S group maintained interpersonal communication with individuals other than parents during the year prior to the arrest (*n* = 13; 32%) than in the other two groups.

### History of behavioral problems (Table 4)

**Table 4:** History of behavioral problems

	**Shoplifting (S) Group (*n*** **= 41)**	**Drug offense (D) Group (*n*** **= 14)**	**Control (C) Group (*n*** **= 34)**	**TS**	***p***
	***n *** **(%)**	***n *** **(%)**	***n *** **(%)**		
Substance abuse					
Illicit drug use	0 (0^a^)	14 (100^b^)	21 (62^c^)	50.3	<0.001
Organic solvent	0 (0^a^)	13 (93^b^)	13 (38^c^)	40.9	<0.001
Severe alcoholism	2 (5^a^)	7 (50^b^)	14 (41^b^)	14.8	<0.001
Sexual promiscuity	2 (5^a^)	6 (43^b^)	15 (44^b^)	14.7	<0.001
Self-harm	6 (15^a^)	11 (79^b^)	26 (76^b^)	31.0	<0.001
Shoplifting rate in lifetime	41(100)	14 (100)	20 (59)	-	-
Previous to eating disorder	3 (7)	13 (93)	-	**	<0.001
Prosecution history	41 (100^a^)	4 (29^b^)	15 (75^c^)	33.7	<0.001
Items were limited to food	31 (76^a^)	0 (0^b^)	1 (5^b^)	47.4	<0.001
Imprisonment for shoplifting	41 (100)	0 (0)	2 (10)	75.9	<0.001
Economically distressed	4 (10)	-	2 (100)	-	-
Previous imprisonment	7 (17)	-	1 (5)	-	-
Other prosecution history of drug-related offenses	0 (0^a^)	14 (100^b^)	14 (41^c^)	45.9	< 0.001
Other crimes*	2 (5^a^)	8 (57^b^)	19 (56^b^)	23.3	< 0.001
Past imprisonment for other than shoplifting	0 (0)	5 (36)	13 (38)	16.2	< 0.001

Significance level: *p* < 0.05.

Significant differences between two or among three groups: Statistical tests used were the *χ*^2^ test, Yates exact test (if *n* < 10) or Fisher’s exact test for dichotomous variables. Post-hoc analyses for significant differences (*χ*^2^ test: *p* < 0.05) are indicated by superscript letters “a” and “b.” Groups sharing a letter did not significantly differ. Degree of freedom (df): *χ*^2^ test for all three groups, 2; *χ*^2^ test for two groups, 1. *Other crimes include murder, assault, fraud, and theft other than shoplifting. **Data could not be detected from Fisher’s exact probability.

Even when patients with eating disorders were divided into the groups described in the Method section, only one patient had a history of substance use among the patients incarcerated for shoplifting. Patients in the S group were also less likely to be sexually promiscuous (*n* = 2; 5%) or self-harming (*n* = 6; 15%) than were patients in the D and C groups.

Although the proportion of patients with a history of shoplifting was >50% in the D (*n* = 14; 100%) and C (*n* = 20; 59%) groups, very few of these patients had actually been incarcerated for this crime in the past (*n* = 0 and 2, respectively). Only three patients in the S group had engaged in shoplifting prior to the onset of their eating disorder. The shoplifted items were typically limited to food in the S group (*n* = 31; 76%). Very few patients in the S group were prosecuted for other crimes (*n* = 2; 5%), and none had been incarcerated for such offenses.

### Clinical observations of behavioral problems (Table 5)

**Table 5:** Clinical observations of behavioral problems

	**Shoplifting (S) Group (*n*** **= 41)**	**Drug offense (D) Group (*n*** **= 14)**	**Control (C) Group (*n*** **= 34)**	**TS**	***p***
	***n *** **(%)**	***n *** **(%)**	***n *** **(%)**		
Violent language	10 (24)	6 (43)	14 (41)	1.9	0.38
Frequent requests for drugs	5 (12^a^)	11 (79^b^)	8 (23^a^)	20.3	<0.001
Self-harm	3 (7^a^)	6 (43^b^)	19 (56^b^)	18.7	<0.001
Food refusal	27(66^a^)	3 (21^b^)	11 (32^b^)	10.2	0.006
Purging	28 (68^a^)	11 (79^a^)	1 (3^b^)	35.8	< 0.001
Falsifying dietary intake amount	32 (78^a^)	4 (29^b^)	0 (0^c^)	44.4	< 0.001
Food hoarding	23 (^a^)56	2 (14^b^)	0 (0^b^)	27.2	<0.001
Excessive exercise	19 (46^a^)	0 (0)	0 (0)	24.4	<0.001

Significance level: *p* < 0.05.

Post-hoc analyses for significant differences (*χ*^2^ test: *p* < 0.05) are indicated by superscript letters “a” and “b.” Groups sharing a letter did not significantly differ.

Behavioral problems during the clinical course were classified as positive if the behavior continued for ≥4 weeks at a frequency threshold of almost every day or more. Statistical tests used were the *χ*^2^ test for three groups, Yates exact test (if *n* < 10). Degrees of freedom = 2.

The S group rarely resorted to disruptive behavior, such as frequent drug requests (*n* = 5; 12%) or self-harm (*n* = 3; 7%). Although the incidence of purging was not significantly different between the S and D groups, patients in the S group more frequently engaged in food refusal (*n* = 27; 66%), falsification of dietary intake amounts (*n* = 32; 78%), food hoarding (*n* = 23; 56%), and excessive exercise (*n* = 19; 46%) than did patients in the D group.

## Discussion

Most patients in the S group did not exhibit impulsive or antisocial behavior. Their education level was significantly higher, and they had a steadier occupational history than did patients in the other groups. This indicates that patients in the S group essentially had higher adaptive societal functioning without antisocial features. However, patients in the S group had long histories of eating disorders that were associated with their higher age, and they had lower body weights at the time of their last arrest than did patients in the other groups. Moreover, most had previously dropped out of treatment and/or ended interpersonal relationships before their latest incarceration. Food refusal and purging were symptoms common to both eating disorder groups during hospitalization, but falsification of dietary intake amounts, food hoarding, and excessive exercise were observed more frequently in the S group.

### Impulsivity and bulimia

In general, shoplifting by patients with eating disorders has been viewed in conjunction with impulsive behaviors, addictive behaviors, antisocial features, BN, and/or severe clinical symptoms [11-19]. However, it should be noted that most patients in the S group had almost no impulsivity, addiction, antisocial features, or BN problems either in the past or during current therapy. These patients were raised in home environments without antisocial features, poverty, or family disintegration; furthermore, they were highly educated. Although they initially acquired vocational skills and were socially adapted, they ultimately progressed to social isolation, which was often influenced by their prolonged and severe eating disorders and poor adherence to treatment. Although the crossover rate to bulimia from anorexia was reportedly 34% in one study [26], we identified only 7 patients with prior BN as opposed to 12 with AN-R. We strongly believe that binge–purge behavior is not a prerequisite for repeated shoplifting behavior among patients with eating disorders.

### Severity of eating disorders, OC traits, and shoplifting

Patients in the S group were significantly older than patients in the other groups. Almost all patients in the S group had a prolonged history of eating disorders; that is, their eating disorder could not be resolved before middle age. Patients in the S group exhibited prolonged histories of severe eating disorders and demonstrated extremely low BMIs at the time of imprisonment. Under such an illness history, behavioral problems such as food refusal, food hoarding, falsification of dietary intake, and excessive exercise were more prominent in the S group. This is consistent with previous reports of the association between shoplifting and severe eating disorders [10,12,23,24]. This may represent a paradoxical pathology where the desire for food is abnormally amplified because of abnormal dietary restrictions and longstanding starvation, resulting in increasingly relentless efforts to gather food. One study suggested that women who have recovered from AN fail to differentiate between reward and punishment and that they are less able to modulate their affective responses to stimuli [27].

A primary diagnosis of OCD with comorbid eating disorders may be associated with increased clinical severity [28]. Although the rate of comorbid OCD was not high among patients in the S group, the frequency of eating-related abnormal behaviors was high, including food hoarding, falsification of dietary intake, and excessive exercise. Food hoarding is a particularly important violation in prisons and may lead to lost parole opportunities. Despite this fact, many patients could not stop their violation behaviors even after being incarcerated. It is possible that these behaviors were an expression of the OC characteristics of patients in the S group. It has also been suggested that all eating disorders can be considered to lie along a spectrum of disorders with varying degrees of OC and impulsive traits [29]. We assumed that the AN-R observed in the S group represented the obsessive pole of this spectrum, whereas AN of the binge–purge type and BN represented the impulsive pole [29,30]. Excessive exercise, which was more frequently observed in the S group, has been considered to be a compulsive behavior and indicates greater eating disorder severity [31-35]. These findings suggest that repeated shoplifting in patients with eating disorders is an OC trait.

### Unstable eating disorder treatment history

Most patients in the S group had previously undergone eating disorder treatment (*n* = 29; 69%), but their adherence to treatment had been poor. Although these patients had severe behavioral problems over many years, most had unstable treatment histories or had dropped out of treatment. It is often difficult to determine whether interventional treatment should be administered, even when abnormalities and risks have been objectively assessed. This is because there is no clear consensus on the extent to which behavioral problems and autonomy should be respected in patients with eating disorders. This complicated situation might be supported by the fact that the patients’ BMIs at the time of arrest did not meet the requirements for immediate compulsory physical treatment despite the fact that they were experiencing a social crisis. In addition, persuading such patients to accept involuntary interventional treatment can be extremely difficult. Compulsory treatment is often determined according to the patient’s physical condition. However, the debate is very difficult because it is unknown whether compulsory treatment can be enforced among patients with eating disorders who are at risk of engaging in repeated shoplifting [36-38]. In the field of eating disorders, we believe that some behavioral problems crossing over into illegal territory should be considered to be as high-risk as a physical crisis in such patients.

### Therapeutic environment in medical prison

We have demonstrated that stable treatment without interruption or dropout may potentially improve serious conditions and behaviors associated with eating disorders before deterioration of the patient’s criminal behavior. We believe that such medical rescue was necessary for the particular patients with eating disorders in this study to help them avoid potential social ruin or recurrent imprisonment associated with their additional behavioral problems. We believe that various types of bias are possible among patients in the specific medical prison environment. However, under circumstances such as those described in the Background section of this report, we do not believe that patients generally want to stay longer in medical prison. Patients in medical prisons usually do not have the opportunity for parole. General prisons are not usually distant from facilities to which individuals convicted of misdemeanors are sent, but our medical prison is in Tokyo; thus, the opportunities for visitation by the patients’ families are likely to be reduced.

Finally, there are fewer types of recreational activities available in medical prisons than in general prisons because recreational activities are generally rewards for labor. For these and other reasons, relatively higher numbers of patients want to return to general prison than to medical prison. In group therapy, patients in the process of recovery often advise patients who have just arrived at the medical prison. Such advice is passed from patient to patient for many years.

We believe that the results of this study are limited to the specific types of patients and the particular setting described herein. This was a preliminary investigation because the study topic is an understudied area. This study also had the following limitations. Only patients currently in an extreme anorexic state were included. Additionally, patients with eating disorders whose weight loss was less serious and patients with bulimia were not included because they can be managed without risk in general facilities.

## Conclusions

Although our results are limited to medical prisons, they show that most patients with AN who are incarcerated for repeated shoplifting and have no history of drug use do not display antisocial characteristics or impulsivity. The psychopathology of severe eating disorders in these patients with eating disorders who were incarcerated only for shoplifting is possibly related to OC behaviors. It is possible that repeated shoplifting may be observed if the patient’s history of eating disorder is prolonged, severe, and untreated, even in the absence of antisocial features. Stable treatment that provides consistent help without providing an opportunity to drop out should be considered at an earlier stage for patients with serious eating disorders, particularly if they show a tendency toward shoplifting. Further research using a multidisciplinary approach is necessary to fully elucidate the relationship between shoplifting and eating disorders.

## Abbreviations

AN: Anorexia nervosa; AN-R: Restricting-type anorexia nervosa; ANOVA: Analysis of variance; BMI: Body mass index; BN: Bulimia nervosa; ED: Eating disorder; OC: Obsessive–compulsive; OCD: Obsessive–compulsive disorder.

## Competing interests

The authors declare that they have no competing interests.

## Authors’ contributions

TA designed the study. TA, MS, and TN carried out the data collection. TA and YO equally contributed to the manuscript; both were also involved in the data analysis and writing of the manuscript. All authors read and approved the final manuscript.

## Authors’ information

TA, SM, and TN were psychiatrists at the Hachioji Medical Prison and researchers at Nippon Medical School. After retiring from the Hachioji Medical Prison in 2009, TN became a professor of psychology at Nippon Medical School. YO is a professor of psychiatry at Nippon Medical School. The sponsor of this study played no role in the study design, collection, analysis, and interpretation of data, writing of the report, or decision to submit the paper for publication. The corresponding author has full access to all data in the study and takes final responsibility for the decision to submit the paper for publication.

## Pre-publication history

The pre-publication history for this paper can be accessed here:

http://www.biomedcentral.com/1471-244X/14/169/prepub

## References

[B1] Birmingham CL Hlynsky J Russell B Gritzner S Pilot treatment program for shoplifting in eating disorders Eat Weight Disord 2005 10 105 108 10.1007/BF03327501 16682864

[B2] Casper RC Eckert ED Halmi KA Goldberg SC Davis JM Bulimia: its incidence and clinical importance in patients with anorexia nervosa Arch Gen Psychiatr 1980 37 1030 1035 10.1001/archpsyc.1980.01780220068007 6932187

[B3] Norton KRW Crisp AH Bhat AV Why do some anorexics steal? Personal, social and illness factors J Psychiatr Res 1985 19 385 390 10.1016/0022-3956(85)90045-7 4045752

[B4] Crisp AH Anorexia nervosa at normal body weight!—The abnormal normal weight control syndrome Int J Psychiatr Med 1981 11 203 233 10.2190/JY71-R8LJ-TUND-9KP4 7309391

[B5] Garfinkel PE Moldofsky H Gamer DM The heterogeneity of anorexia nervosa: Bulimia as a distinct subgroup Arch Gen Psychiatr 1980 37 1036 1040 10.1001/archpsyc.1980.01780220074008 6932188

[B6] Takagi S Oomori M Hamanaka S Misono Y Forensic problems of the shoplifting by eating disorders Jpn J Clin Psychiatr 2008 37 1421 1427

[B7] Suzuki K Takeda A The clinical characteristics of and treatment for habitual shoplifting in patients with eating disorders Seishin Igaku 2010 52 647 654

[B8] Yoshinaga T Abe M Asami T Odagiri M Nishiguchi Y Problem in treating patients with eating disorders in correctional facilities in Japan J Correctional Med 2012 60 54 90

[B9] Blanco C Grant J Petry NM Simpson HB Alegria A Liu SM Hasin D Prevalence and correlates of shoplifting in the United States: results from the National Epidemiologic Survey on Alcohol and Related Conditions (NESARC) Am J Psychiatr 2008 165 905 913 10.1176/appi.ajp.2008.07101660 18381900 PMC4104590

[B10] Baum A Goldner EM The relationship between stealing and eating disorders: a review Harv Rev Psychiatr 1995 3 210 221 10.3109/10673229509017187 9384949

[B11] Fichter MM Quadflieg N Rief W Course of multi-impulsive bulimia Psychol Med 1994 24 591 604 10.1017/S0033291700027744 7991741

[B12] Lacey JH Self-damaging and addictive behavior in bulimia nervosa. A catchment area study Br J Psychiatr 1993 163 190 194 10.1192/bjp.163.2.190 8075910

[B13] Lacey JH Evans CDH The Impulsivist: a multi-impulsive personality disorder Br J Addict 1986 81 641 649 10.1111/j.1360-0443.1986.tb00382.x 3539167

[B14] Matsunaga H Kiriike N Iwasaki Y Miyata A Matsui T Nagata T Yamagami S Kaye WH Multi-impulsivity among bulimic patients in Japan Int J Eat Disord 2000 27 348 352 10.1002/(SICI)1098-108X(200004)27:3<348::AID-EAT13>3.0.CO;2-F 10694722

[B15] Peñas-Lledó E Vaz FJ Ramos MI Waller G Impulsive behaviors in bulimic patients: relation to general psychopathology Int J Eat Disord 2002 32 98 102 10.1002/eat.10039 12183951

[B16] Wiederman MW Pryor T Multi-impulsivity among women with bulimia nervosa Int J Eat Disord 1996 20 359 365 10.1002/(SICI)1098-108X(199612)20:4<359::AID-EAT3>3.0.CO;2-M 8953323

[B17] Nagata T Kawarada Y Kiriike N Iketani T Multi-impulsivity of Japanese patients with eating disorders: primary and secondary impulsivity Psychiatr Res 2000 94 239 250 10.1016/S0165-1781(00)00157-8 10889288

[B18] Myers TC Wonderlich SA Crosby R Mitchell JE Steffen KJ Smyth J Miltenberger R Is multi‒impulsive bulimia a distinct type of bulimia nervosa: psychopathology and EMA findings Int J Eat Disord 2006 39 655 661 10.1002/eat.20324 16927382

[B19] Fullerton DT Wonderlich SA Gosnell BA Clinical characteristics of eating disorder patients who report sexual or physical abuse Int J Eat Disord 1995 17 243 249 10.1002/1098-108X(199504)17:3<243::AID-EAT2260170305>3.0.CO;2-Z 7773261

[B20] Selby EA Bulik CM Thornton L Brandt HA Crawford S Fichter MM Halmi KA Jacoby GE Johnson CL Jones I Kaplan AS Mitchell JE Nutzinger DO Strober M Treasure J Woodside DB Kaye WH Joiner TE Jr Refining behavioral dysregulation in borderline personality disorder using a sample of women with anorexia nervosa Personal Disord 2010 1 4 250 257 22448667 10.1037/a0019313 PMC4688899

[B21] Goldner EM Geller J Birmingham CL Remick RA Comparison of shoplifting behaviors in patients with eating disorders, psychiatric control subjects, and undergraduate control subjects Can J Psychiatr 2000 45 471 475 10.1177/070674370004500508 10900528

[B22] Nozoe S-I Soejima Y Yoshioka M Naruo T Masuda A Nagai N Tanaka H Clinical features of patients with anorexia nervosa: assessment of factors influencing the duration of in-patient treatment J Psychosom Res 1995 39 271 281 10.1016/0022-3999(94)00141-Q 7636771

[B23] Krahn DD Nairn K Gosnell BA Drewnowski A Stealing in eating disordered patients J Clin Psychiatr 1991 52 112 115 2005074

[B24] Vandereycken W Houdenhove VV Stealing behavior in eating disorders: characteristics and associated psychopathology Compr Psychiatr 1996 37 316 321 10.1016/S0010-440X(96)90012-7 8879905

[B25] Brener N Eating disorders and shoplifting–a study in two settings Med Sci Law 1993 33 223 226 8366784 10.1177/002580249303300307

[B26] Eddy K Dorer D Franko D Tahilani K Thompson-Brenner H Herzog D Diagnostic crossover in anorexia nervosa and bulimia nervosa: implications for DSM-V Am J Psychiatr 2008 165 245 250 10.1176/appi.ajp.2007.07060951 18198267 PMC3684068

[B27] Wagner A Aizenstein H Venkatraman VK Fudge J May JC Mazurkewicz L Frank GK Bailer UF Fischer L Nguyen V Carter C Putnam K Kaye WH Altered reward processing in Women recovered from anorexia nervosa Am J Psychiatr 2007 164 1842 1849 10.1176/appi.ajp.2007.07040575 18056239

[B28] Sallet PC de Alvarenga PG Ferrão Y de Mathis MA Torres AR Marques A Hounie AG Fossaluza V do Rosario MC Fontenelle LF Petribu K Fleitlich-Bilyk B Eating disorders in patients with obsessive–compulsive disorder: prevalence and clinical correlates Int J Eat Disord 2010 43 315 325 19424977 10.1002/eat.20697

[B29] Claes L Nederkoorn C Vandereycken W Guerrieri R Vertommen H Impulsiveness and lack of inhibitory control in eating disorders Eat Behav 2006 7 196 203 10.1016/j.eatbeh.2006.05.001 16843221

[B30] McElroy SL Phillips KA Keck PE Obsessive compulsive spectrum disorders J Clin Psychiatr 1994 55 33 51 7961531

[B31] Bratland-Sanda S Sundgot-Borgen J Rø Ø Rosenvinge JH Hoffart A Martinsen EW Physical activity and exercise dependence during inpatient treatment of longstanding eating disorders: an exploratory study of excessive and non-excessive exercisers Int J Eat Disord 2010 43 266 273 19839057 10.1002/eat.20769

[B32] Davis C Kaptein S Anorexia nervosa with excessive exercise: a phenotype with close links to obsessive-compulsive disorder Psych Res 2006 142 209 217 10.1016/j.psychres.2005.11.006 16697469

[B33] Adkins EC Keel PK Does “excessive” or “compulsive” best describe exercise as a symptom of bulimia nervosa? Int J Eat Disord 2005 38 24 29 10.1002/eat.20140 15991218

[B34] Bewell-Weiss CV Carter JC Predictors of excessive exercise in anorexia nervosa Compr Psychiatr 2010 51 566 571 10.1016/j.comppsych.2010.03.002 20965301

[B35] Johnston O Reilly J Kremer J Excessive exercise: from quantitative categorization to a qualitative continuum approach Eur Eat Disord Rev 2011 19 237 248 10.1002/erv.970 21584916

[B36] Holm JS Brixen K Andries A Hørder K Støving RK Reflections on involuntary treatment in the prevention of fatal anorexia nervosa: a review of five cases Int J Eat Disord 2012 45 93 100 10.1002/eat.20915 21344467

[B37] Ramsay R Ward A Treasure J Russell G Compulsory treatment in anorexia nervosa. Short-term benefits and long-term mortality Br J Psychiatr 1999 175 147 153 10.1192/bjp.175.2.147 10627797

[B38] Watson TL Bowers WA Andersen AE Involuntary treatment of eating disorders Am J Psychiatr 2000 157 1806 1810 10.1176/appi.ajp.157.11.1806 11058478

